# Molecular docking analysis of N-substituted Oseltamivir derivatives with the SARS-CoV-2 main protease

**DOI:** 10.6026/97320630016404

**Published:** 2020-05-31

**Authors:** Assia Belhassan, Samir Chtita, Hanane Zaki, Tahar Lakhlifi, Mohammed Bouachrine

**Affiliations:** 1 Molecular Chemistry and Natural Substances Laboratory, Faculty of Science, Moulay Ismail University of Meknes, Morocco; 2 Laboratory of physical chemistry and materials, Faculty of science Ben M'Sik, Hassan II University of Casablanca, Casablanca, Morocco; 3 EST Khenifra, Sultan Moulay Sliman University, Benimellal, Morocco

**Keywords:** Coronavirus, COVID-19, SARS-CoV-2, oseltamivir, H5N1, molecular docking

## Abstract

The identification of chemotherapeutic drugs against Novel Coronavirus (2019-nCoV) is a significant requirement due to the rapid rise in deaths due to Corona Viral Infection all around the world. Therefore, it is of interest to document the molecular docking analysis data of 32 N-substituted Oseltamivir derivatives inhibitors of influenza virus H5N1 with the Novel Coronavirus main protease (2019-nCoV). We describe the optimal binding features of Oseltamivir derivatives with the SARS-Cov-2 main protease (Code PDB: 6LU7) for further consideration.

## Background

2019 novel coronavirus (SARS-CoV-2) is reported in December 2019 in Wuhan (China). This infection has spread in the majority of countries and caused until early April 2020, 823 626 confirmed cases and 40 598 deaths according to the world health organization [1-3]. SARS-CoV-2 who causes COVID-19 is a member of Beta-corona viruses like the Severe Acute Respiratory Syndrome coronavirus (SARS-CoV) and the Middle-East Respiratory Syndrome coronavirus (MERSHCoV) [4]. So far no drugs or vaccine against this virus has been reported, due to the long time of producing new medicines, repurposing medications may be the only way to fight this unexpected pandemic. Among the drugs proposed as antiviral agents of COVID-19, we selected Oseltamivir [2,5]. To date, oseltamivir has been the first choice as an effective treatment for infections with influenza A and B, and has been widely used since its approval in 1999 [6]. Many papers reported the effect of chloroquine and hydroxy chloroquine in the treatment of COVID-19 [2,7- 9], today these two drugs are used in different hospitals around the world as antiviral treatment of COVID-19 disease caused by SARS-CoV-2 virus [10,11]. Based on this effect the study of interactions of chloroquine, hydroxy chloroquine and thirty-two N-substituted Oseltamivir derivatives in the active site of SARS-CoV-2 main protease recently crystallized are recommended. Therefore, it is of interest to document the molecular docking analysis data of 32 N-substituted Oseltamivir derivatives inhibitors of influenza virus H5N1 with the Novel Coronavirus main protease (2019-nCoV) with known structure (Code PDB: 6LU7).

## Materials and Methods

### Data set:

**Figure 1:**
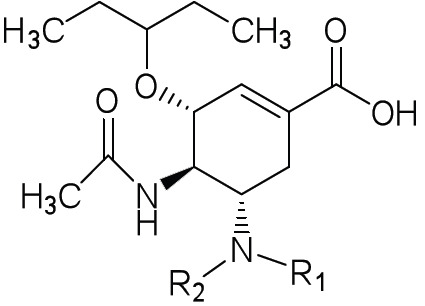
General chemical structure for oseltamivir derivatives with varying R groups

The studied compounds were evaluated against Novel Coronavirus (SARS-CoV-2 main protease) (Figure 1andTable 1). The chemical compounds reported as potent avian influenza virus H5N1 inhibitors from N-substituted Oseltamivir derivatives were taken from literature [6].

### Minimization:

Each structure of 32 moleculesis sketched with Gauss View 05 program [12],and optimized by DFT approach performed with Gaussian 09 program package [13] using the hybrid functional B3LYP [14] combining the Becke's three-parameter and the Lee-Yang-Parr exchange-correlation functional employing the 6-31G(d) basis set in gas phase [15]. The geometry of the compounds was determined by optimizing all geometrical variables with no symmetry constraint [16].

### Molecular Docking:

Molecular docking carried out to determine binding affinity and predict the intermolecular interactions of molecules in targets (protein or enzyme). We performed a docking of 32 potent avian influenza virus H5N1 inhibitors based N-substituted Oseltamivir derivatives in the binding pocket of SARS-CoV-2 main protease (pdb code 6LU7) [17]. The docking study was carried out with two programs; Autodock Vina [18] and Autodock tools 1.5.6 [19]. The crystallographic structure of COVID-19 main protease (pdb code 6LU7) is imported into "work space" of Discovery Studio 2016 program [20] to obtain the binding site [21], and molecules of water are removed. The active site has been determined and it corresponds to the coordinates: x= -26.283, y = -12.599 and z=58.965. The grid size was set at 40x40x40 xyz points with grid spacing of 1 Å to cover the folic acid binding site in the enzyme and was generated by using the co-crystallized ligand (N3) as the center for docking. For Autodock Vina study, an extended PDB format, termed PDBQT, is used for cordonnante files, which includes atomic partial charges and atom types. Torsion angles were calculated to assign the flexible and non-bonded rotation of molecules.

## Results and discussion:

Molecular docking was performed to find types of interactions and the binding affinity of studied molecules in the target. 32 different molecules have been evaluated for their affinity against the SARS-CoV-2 main protease (pdb code 6LU7). The results are presented in (Table 2).

**Figure 2:**
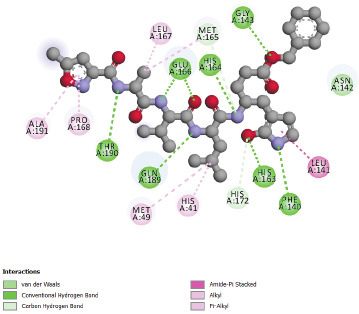
Interactions between inhibitor N3 (co-crystallized ligand) and SARS-CoV-2 main protease

**Figure 3:**
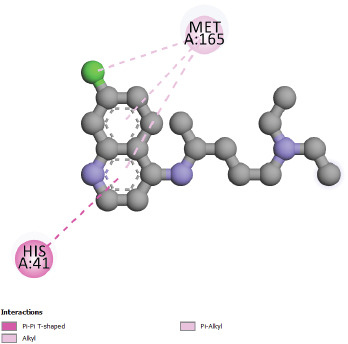
Interactions between Chloroquine and SARS-CoV-2 main protease

The best energies of interaction with the SARS-CoV-2 main protease (pdb code 6LU7) (lowest energy level) are observed for compounds N° 24, 28, 31 and 32 (Table 2). So, these molecules could have more inhibitory potential of the studied enzyme than Chloroquine and Hydroxychloroquine. The structures of the molecules that have the best affinity in the binding site of SARS-CoV-2 main protease indicate that the present of the cycle and/or Fluorine atoms in the structure increase predicted inhibitory potential of these molecules against studied enzyme. The co-crystallized ligand taken from the crystal structure of studied enzyme is re-docked into the active site. It could be seen from Figure 2 that they are present of Conventional Hydrogen Bonds interactions with Glu 166, His 164, Gly 143, Thr 190, Gln 189, His 163 and Phe 140 residues, amide-π Staked interactions with Leu 141 residue, Alkyl and/or π-Alkyl interactions with Leu 167, Ala 191, Met 167 and Pro 168, Met 49 and His 41 residues, Carbon Hydrogen Bond with Met 165 and His 172 residues and Van der Waals interaction with Asn142. The interaction results of Chloroquine and SARS-CoV-2 main protease 6LU7 (Figure 3), shows π-π T-Shaped bond with His 41 residue, π-alkyl and alkyl interactions with Met165 residue.

**Figure 4:**
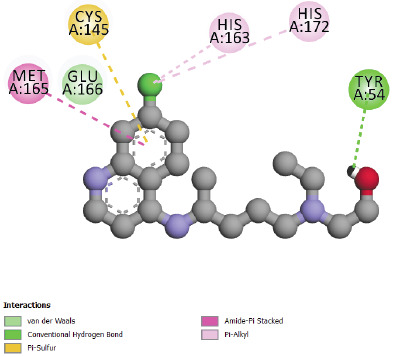
Interactions between Hydroxychloroquine and SARS-CoV-2 main protease

**Figure 5:**
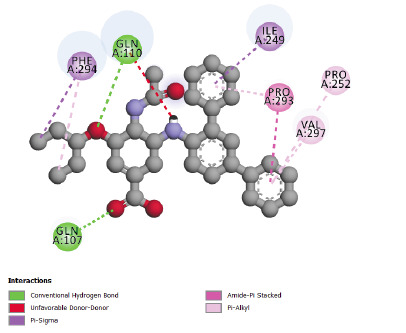
Interactions between compound N°31 and SARS-CoV-2 main protease

**Figure 6:**
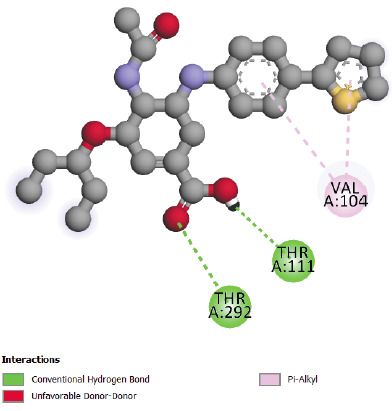
Interactions between compound N°32 and SARS-CoV-2 main protease

**Figure 7:**
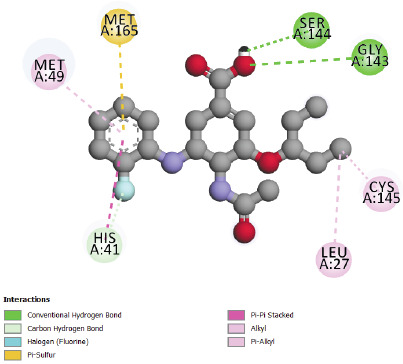
Interactions between compound N°24 and the SARS-CoV-2 main protease

Docking study of Hydroxychloroquine in SARS-CoV-2 main protease 6LU7 (Figure 4) shows more interactions (van der Waals, p-alkyl, amide-p stacked, p-sulfure and hydrogen bond interaction) in comparison with Chloroquine. The presence of hydrogen bonding and Van Der Walls interactions could give to Hydroxychloroquine a pharmacological importance compared to Chloroquine, actually hydrogen bonds play a major role in the pharmacological effect of ligands. The interaction results of compounds N° 24, 31 and 32 in SARS-CoV-2 main protease 6LU7 (Figure 5,Figure 6 and Figure 7) shows more type and number of interactions in comparison with Chloroquine and Hydroxychloroquine of studied enzyme (p-alkyl, alkyl, Amide-p Stacked, Fluorine, p-Sulfure, p-Sigma, Carbon-hydrogen bond and Hydrogen Bond interaction). So these compounds (Molecules N° 24, 28, 31 and 32) could have more inhibitory potential of the studied enzyme than Chloroquine and Hydroxychloroquine, because of their different interactions and the best affinity in the binding pocket of SARS-CoV-2 main protease (6LU7). The number can explain this affinity and type of bonds noticed in these molecules, indeed the presence of hydrogen bonds shows an important potential pharmacological effect, by the inhibition of SARS-CoV-2 main protease 6LU7. The inhibition of this protein will induce the inhibition of viral replication; these results show that these molecules could be interesting in the clinical management of COVID-19.

## Conclusions:

We describe the structural binding features of Oseltamivir derivatives with the SARS-Cov-2 main protease (Code PDB: 6LU7) for further consideration.

## Figures and Tables

**Table 1:** Chemical groups representing R for 32 Oseltamivir derivatives (see Figure 1) reported as potent avian influenza

N°	R1	R2	N°	R1	R2
1	Thiophen-2-yl	Amino(imino-)methyl	17	Propan-2-yl	H
2	Phenyl	Amino(imino-)methyl	18	Thiophen-2-yl	H
3	4-(propan-2-yl)phenyl	Amino(imino-)methyl	19	Phenyl	H
4	2-methoxyphenyl	Amino(imino-)methyl	20	2-methoxyphenyl	H
5	[1, 1'-biphenyl]-4-yl	Amino(imino-)methyl	21	2-hydroxyphenyl	H
6	Ethyl	Amino(imino-)methyl	22	2-chlorophenyl	H
7	Propan-2-yl	Amino(imino-)methyl	23	2-bromophenyl	H
8	Butyl	Amino(imino-)methyl	24	2-fluorophenyl	H
9	Propyl	Amino(imino-)methyl	25	2-phenylethenyl	H
10	2-methylpropyl	Amino(imino-)methyl	26	[1, 1'-biphenyl]-4-yl	H
11	Pentan-3-yl	Amino(imino-)methyl	27	2-phenylethyl	H
12	2-phenylethyl	Amino(imino-)methyl	28	2-[(furan-2-yl)methoxy]phenyl	H
13	H	H	29	2-(ethenyloxy)phenyl	H
14	Amino(imino-)methyl	H	30	3-chloro[1,1'-biphenyl]-4-yl	H
15	Pentan-3-yl	H	31	[1,1:3,1-terphenyl]-4-yl	H
16	Propyl	H	32	4-(thiophen-2yl)phenyl	H

**Table 2:** The results of the docking study: the best pose conformation ordered by their binding affinities.

N°	Affinity (Kcal/mol)	N°	Affinity (Kcal/mol)	N°	Affinity (Kcal/mol)
					
31	-7.8	12	-6.7	4	-6.1
32	-7.7	22	-6.7	9	-6.1
24	-7.4	19	-6.6	11	-6.1
28	-7.4	10	-6.4	15	-6.1
26	-7.3	14	-6.4	17	-6.1
30	-7.3	29	-6.4	21	-6.1
3	-7.1	6	-6.3	8	-6
5	-7.1	2	-6.2	13	-6
20	-7	7	-6.2	27	-5.9
23	-6.8	18	-6.2	16	-5.3
25	-6.8	1	-6.1	Chloroquine	-5.7
				Hydroxychloroquine	-6.5
